# MISTIC-fusion proteins as antigens for high quality membrane protein antibodies

**DOI:** 10.1038/srep41519

**Published:** 2017-02-02

**Authors:** Natalia Silva Alves, Susanne Adina Astrinidis, Nathalie Eisenhardt, Cornelia Sieverding, Josef Redolfi, Michael Lorenz, Marion Weberruss, Daniel Moreno-Andrés, Wolfram Antonin

**Affiliations:** 1 Friedrich Miescher Laboratory of the Max Planck Society, Spemannstraße 39, 72076 Tübingen, Germany

## Abstract

Lack of high-quality antibodies against transmembrane proteins is a widely recognized hindrance in biomedical and cell biological research. Here we present a robust pipeline for the generation of polyclonal antibodies employing full-length membrane proteins as immunogens to overcome this “antibody bottleneck”. We express transmembrane proteins fused to a MISTIC fragment that enhances expression of eukaryotic membrane proteins in *E. coli.* Purified membrane proteins are used as immunogen for rabbit injection employing standard immunizing protocols. The raised antibodies against membrane proteins of the endoplasmic reticulum and the nuclear envelope, which we use as test cases, function in a wide range of applications and are superior to ones produced against soluble domains as immunogens.

Human cells have a wide variety of integral membrane proteins, which comprise approximately 30% of proteins encoded by the genome^1^,^2^. These proteins play essential roles in membrane trafficking, signal transduction, growth regulation, pathogen invasion and many other cellular processes. Approximately 60% of drugs currently available in the market target membrane proteins^3^,^4^. Thus, there is a constant need in cell biological and biomedical research for tools studying these proteins. Yet, high quality and versatile antibodies, the molecular workhorses of protein research, against transmembrane proteins are difficult to produce. One outstanding challenge is the preparation of integral membrane proteins in sufficient amounts as a prerequisite to generate functional antibodies. Traditionally, this problem has been bypassed by using peptide fragments or small soluble domains of the protein as immunogen at the expense of the antibody quality and overall success rate^5^.

Most membrane proteins do not exist in abundance naturally. Hence, heterologous expression of integral membrane proteins is in most instances a prerequisite for using them as antigens. Expression in yeast, plants, insect and mammalian cells as well as cell free systems have been employed for generation and purification of integral membrane proteins^6^,^7^,^8^,^9^,^10^. These techniques involve relative high costs and the success rate is often unpredictable. Thus, bacteria, most importantly *Escherichia coli*, are the most widely used hosts when attempting production of membrane proteins^11^. This is despite the fact that integral membrane proteins often express in relative low yields as compared to soluble proteins or are found in inclusion bodies which requires denaturing purification protocols.

Recently, a 110 aa membrane associated protein from *Bacillus subtiliis*, MISTIC, has been shown to enhance the expression levels of foreign integral membrane proteins in *E. coli* when generated as fusion proteins^12^,^13^,^14^. Interestingly, unusual for a bacterial membrane protein MISTIC is highly hydrophilic and lacks a detectable signal sequence^14^. It may therefore avoid the bacteria’s translocon machinery to integrate into the bacteria membrane in a *Sec* independent manner. Using this system integral membrane proteins can be expressed in bacteria, extracted from the bacteria membrane and purified under native conditions^15^,^16^,^17^.

Here, we present a simple workflow using MISTIC-fusion proteins for high-yield expression of eukaryotic transmembrane proteins in *E. coli.*, which are used for efficient polyclonal antibody production. After Ni2 + affinity purification the proteins are employed as immunogen for rabbit injection using standard immunizing protocols. The obtained antisera were compared to antisera generated against the soluble domains of the respective proteins in biochemical applications including western blotting, immunoprecipitation and immunofluorescence and were found to be superior in all aspects.

## Results

Isolated soluble domains of an integral membrane protein are often used as antigens for antibody production against membrane proteins. In our experience, the success rate of this strategy is unpredictable. We asked whether this could be improved by employing the full-length protein as antigen thus presenting all potential epitopes to the immune system. For this, we expressed integral membrane proteins as HIS6-tagged MISTIC-fusion proteins in *E. coli* and isolated them by Ni2 + affinity purification in the presence of the detergent Cetyltrimethylammoniumbromid (CTAB)^17^. The purified full-length proteins were used as antigens for injection into rabbits to generate polyclonal antibodies following a standard immunization procedure. To allow comparison to a classical approach we also generated rabbit polyclonal antisera using an isolated soluble domain of an integral membrane protein, in many instances the entire non-transmembrane part of the respective protein, as antigen following the same immunization protocol. We chose as test cases integral membrane proteins of the nuclear envelope and the connected endoplasmic reticulum, a membrane compartment that, also because of its disease relation, attracted recently major attention^18^,^19^.

**Figure 1:**
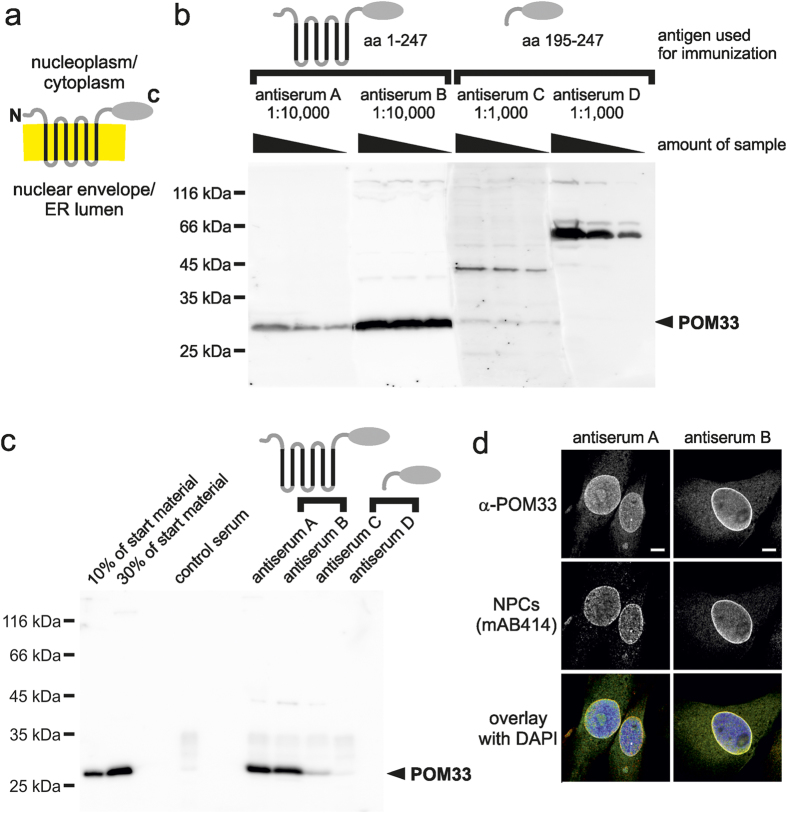
Antisera against full-length POM33 outperform antisera generated against a soluble fragment. (**a**) Schematic representation of POM33. Predicted transmembrane regions are indicated in black, the lipid bilayer in yellow. (**b**) 10 μg, 3 μg and 1 μg of a total membrane fraction from *Xenopus* egg extracts were separated on a 12% SDS-PAGE and analyzed by western blotting using antisera against full-length POM33 (antiserum A and B) in a 1:10,000 dilution and antisera against the C-terminal domain (antiserum C and D) in a 1:1,000 dilution. Molecular size markers as well as the position of POM33 are indicated. Please note that for this analysis the blot membrane was after transfer divided into four parts, which were separately incubated with the indicated antisera. For blot analysis by ECL the parts were realigned and imaged as a whole. (**c**) POM33 was immunprecipitated from a solubilized total membrane fraction from *Xenopus* egg extracts using antisera against full-length POM33 (antiserum A and B) and antisera against the C-terminal domain (antiserum C and D). Antibody bound proteins were eluated with SDS sample buffer and separated together with 10% and 30% of the corresponding starting material on a 12% SDS-PAGE. After western blotting samples were analyzed with α-POM33 antiserum B. (**d**) Immunofluorescence detection of POM33. *Xenopus* S3 cells were fixed with 2% paraformaldehyde and stained with α-POM33 antisera A and B. Samples were co-stained with the nuclear pore complex (NPC) marker mAB414 and DAPI and analyzed by confocal microscopy. Bars: 5 μm.

The first test candidate, POM33 is a multi-pass membrane protein (see Fig. 1a for schematic presentation) of the endoplasmic reticulum and the nuclear envelope^20^. We raised antisera in four rabbits, two were injected with full-length *Xenopus* POM33 expressed and purified as MISTIC-fusion (Fig. 1b, antiserum A and B), and two against the C-terminal domain of the protein (antiserum C and D). Comparative western blotting using *Xenopus* egg extracts shows that both antisera generated against the MISTIC-fusion recognized a protein at the predicted size of 28 kDa whereas only one of the two antisera against the soluble domain recognized the correct protein, however, with a comparative weak signal even at a tenfold higher antiserum concentration (Fig. 1b). In addition, several cross-reactivities were detected when employing the antisera against the soluble domain. Immunoprecipitation experiments testing the four antisera using solubilized *Xenopus* membranes shows that both antisera generated against the MISTIC-fusion efficiently immunprecipitate POM33 whereas only one antisera against the soluble domain was functional albeit much less efficient (Fig. 1c). Both antisera against the full-length protein also performed well in immunofluorescence (Fig. 1d): they stained the nuclear envelope, a typical pattern seen with proteins of nuclear pore complexes, which have been stained with the mouse monoclonal antibody mAB414^21^. In contrast, we did not obtain a specific immunofluorescence signal when employing the two antisera against the soluble domain using a variety of fixation and staining protocols (data not shown). Thus, in all tested applications, both antisera against the full-length POM33 are clearly superior to the antisera generated against the soluble domain.

**Figure 2:**
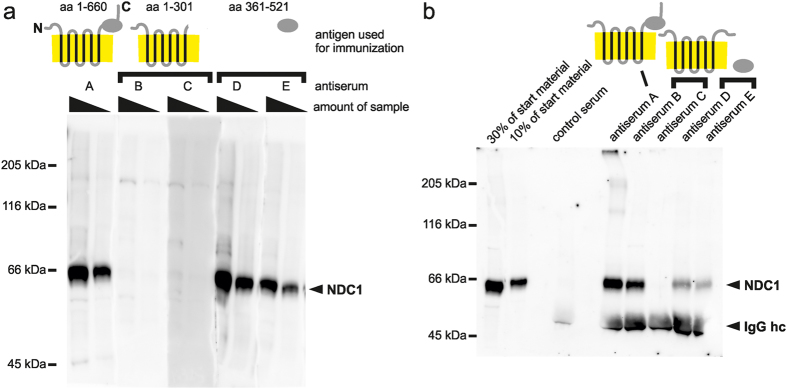
Characterization of antisera against the nuclear pore complex transmembrane protein NDC1. (**a**) 10 μg and 3 μg of a total membrane fraction from *Xenopus* egg extracts were separated on a 8% SDS-PAGE and analyzed by western blotting using antisera against full-length NDC1 (aa 1–660, antiserum A), against the N-terminal part of NDC1 which contains all six predicted transmembrane regions (aa 1–301, antiserum B and C) and against a C-terminal domain (aa 361–521, antiserum D and E, described in ref. 22). All antisera were used in a 1:1,000 dilution. Molecular size markers as well as the position of NDC1 are indicated. (**b**) NDC1 was immunprecipitated from a solubilized total membrane fraction from *Xenopus* egg extracts using antisera against full-length NDC1 (antiserum A), against the N-terminal part of NDC1 (antiserum B and C) and against a C-terminal domain (antiserum D and E). Antibody bound proteins were eluated with SDS sample buffer and separated together with 30% and 10% of the corresponding starting material on a 8% SDS-PAGE. After western blotting samples were analyzed with α-NDC1 antiserum A. Molecular size markers as well as the position of NDC1 and the detected IgG heavy chains (hc) are indicated.

The identical conclusion was drawn when we raised antibodies against two other nuclear envelope membrane proteins. The first, NDC1 is a multispanning membrane protein of nuclear pore complexes with six predicted transmembrane helixes^22^,^23^,^24^. We generated antisera using as antigen the full-length protein (antiserum A) as well as the N-terminal half of the protein which largely comprise all six predicted transmembrane regions (antiserum B and C), both expressed as MISTIC-fusions (Fig. 2). When comparing to two antisera raised against a soluble C-terminal domain and previously characterized^22^ we observed that in western blotting the antiserum against the full-length protein was equivalent or superior to the two antisera raised against a soluble C-terminal. The antisera raised against the N-terminal half of NDC1 did not work efficiently in western blotting which indicates that at least for NDC1 the majority of epitopes is not located in this region. However, one of the antisera raised against the N-terminal half of NDC1 worked efficiently in immunoprecipitation outperforming the antisera against the soluble domain. The antisera against the full-length protein performed even better in immunoprecipitations supporting our notion that antisera generated with MISTIC-fusion proteins are superior to ones against soluble domains of the respective proteins when applied to natively folded target proteins.

**Figure 3:**
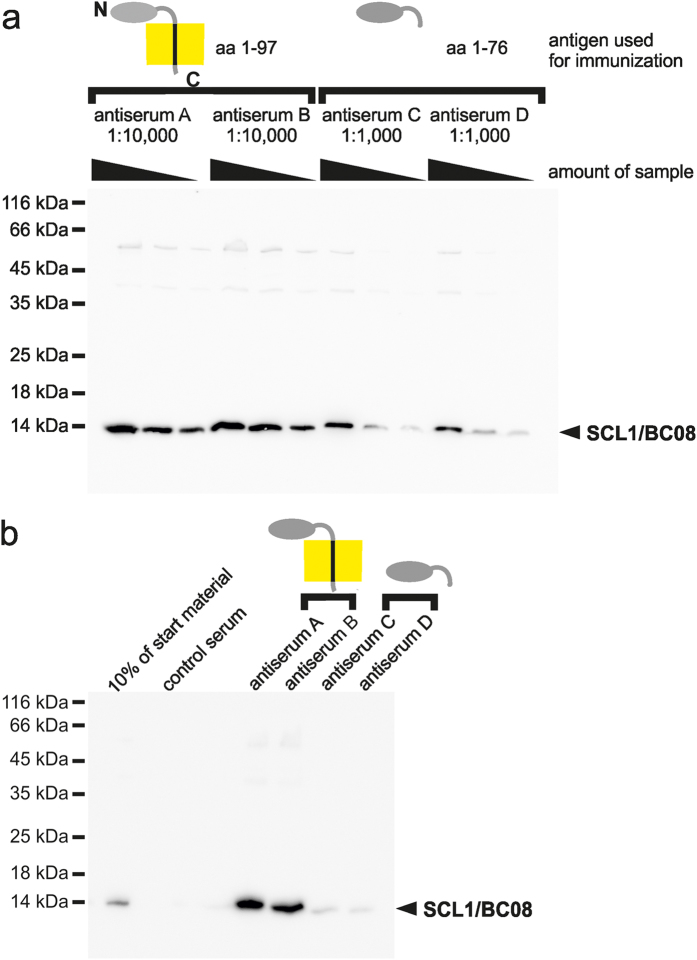
Characterization of antisera against the type II inner nuclear membrane protein SCL1/BC08. (**a**) 10 μg, 3 μg and 1 μg of a total membrane fraction from *Xenopus* egg extracts were separated on a 15% SDS-PAGE and analyzed by western blotting using antisera against full-length SCL1/BC08 (aa 1–97, antiserum A and B) in a 1:10,000 dilution and antisera against the nucleoplasmic domain (aa 1–76, antiserum C and D) in a 1:1,000 dilution. Molecular size markers as well as the position of SCL1/BC08 are indicated. (**b**) SCL1/BC08 was immunprecipitated from a solubilized total membrane fraction from *Xenopus* egg extracts using antisera against full-length SCL1/BC08 (antiserum A and B) and antisera against the nucleoplasmic domain (antiserum C and D). Antibody bound proteins were eluated with SDS sample buffer and separated together with 30% and 100% of the corresponding starting material on a 15% SDS-PAGE. After western blotting samples were analyzed with α- SCL1/BC08 antiserum A.

The next nuclear envelope membrane protein analyzed in detail is SCL1/BC08, a type II single spanning membrane protein of the inner nuclear membrane^25^. Comparative western blotting using *Xenopus* egg extracts shows that both antisera generated against the MISTIC full-length fusion recognized a protein at the predicted size of 14 kDa (Fig. 3a). Antisera raised against the nucleoplasmic part of the protein recognized the same protein, albeit less efficient and only at a higher antisera concentration. Immunoprecipitation experiments testing the four antisera using solubilized *Xenopus* membranes shows that both antisera generated against the MISTIC-fusion efficiently immunprecipitate SCL1/BC08 whereas the antisera against the soluble domain were much less efficient (Fig. 3b). Thus, in all instances tested, the antisera against the full-length proteins outperformed the antisera raised against soluble domains of the respective proteins.

**Figure 4:**
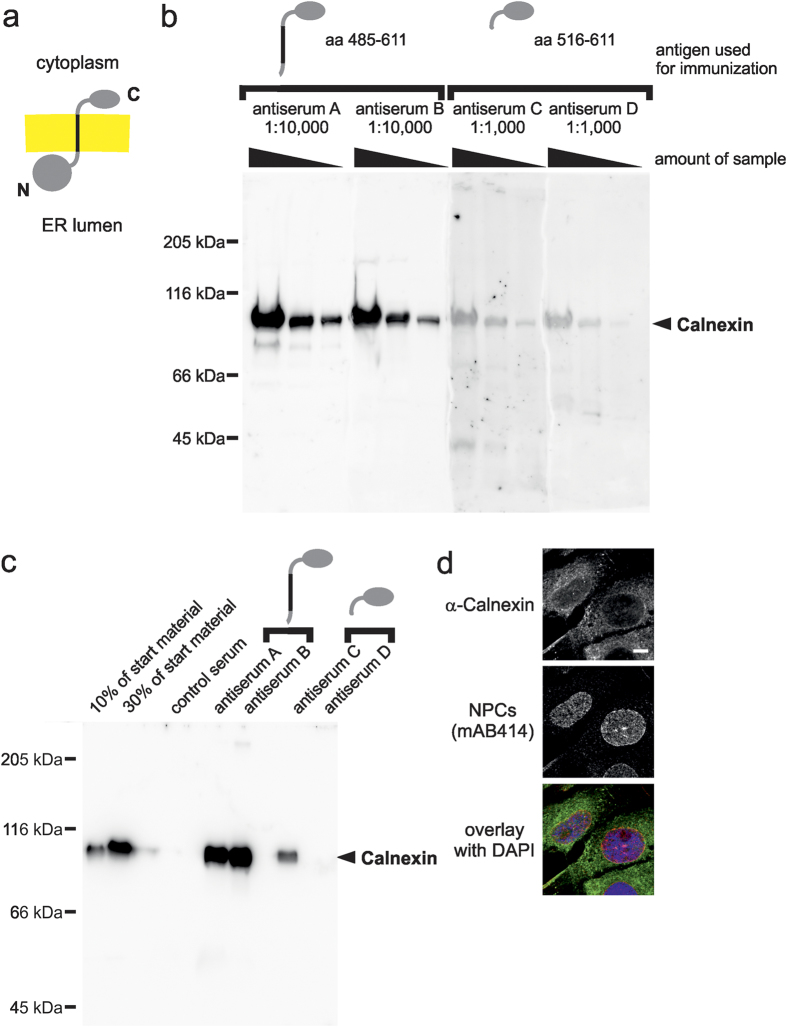
Characterization of Calnexin antisera. (**a**) Schematic representation of Calnexin. The transmembrane region is indicated in black, the lipid bilayer in yellow. (**b**) 10 μg, 3 μg and 1 μg of a total membrane fraction from *Xenopus* egg extracts were separated on a 8% SDS-PAGE and analyzed by western blotting using antisera against a Calnexin fragment including its transmembrane region (aa 485–611, antiserum A and B) in a 1:10,000 dilution and antisera against the C-terminal cytoplasmic domain (aa 516–611 antiserum C and D) in a 1:1,000 dilution. Molecular size markers as well as the position of Calnexin are indicated. (**c**) Calnexin was immunprecipitated from a solubilized total membrane fraction from *Xenopus* egg extracts using antisera against a Calnexin fragment including its transmembrane region (antiserum A and B) and antisera against the C-terminal domain (antiserum C and D). Antibody bound proteins were eluated with SDS sample buffer and separated together with 10% and 30% of the corresponding starting material on a 8% SDS-PAGE. After western blotting samples were analyzed with α-Calnexin antiserum A. (**d**) Immunofluorescence detection of Calnexin. *Xenopus* S3 cells were fixed with 2% paraformaldehyde and stained with antiserum A. Samples were co-stained with the nuclear pore complex (NPC) marker mAB414 and DAPI and analyzed by confocal microscopy. Bar: 5 μm.

Interestingly, the SCL1/BC08 antisera raised against the full-length protein outperformed the antisera developed against the nucleoplasmic domain despite the fact that both antigens are with the exception of 21 aa constituting the a single transmembrane region of SCL1/BC08, identical. This suggests that employing a protein including its membrane domain improves antigen presentation for successful antibody production. In line with this, we observed that the single transmembrane region and the cytoplasmic domain of the endoplasmic reticulum membrane protein Calnexin was a superior antigen for antisera production as the cytoplasmic domain alone (Fig. 4). Antisera raised against aa 465–611 of *Xenopus* Calnexin detected the protein in western blots at a ten-fold higher dilution with a stronger signal than the antisera raised against the cytoplasmic domain (aa 516–611). The former antisera were also superior in immunoprecipitation and immunostainings.

**Figure 5:**
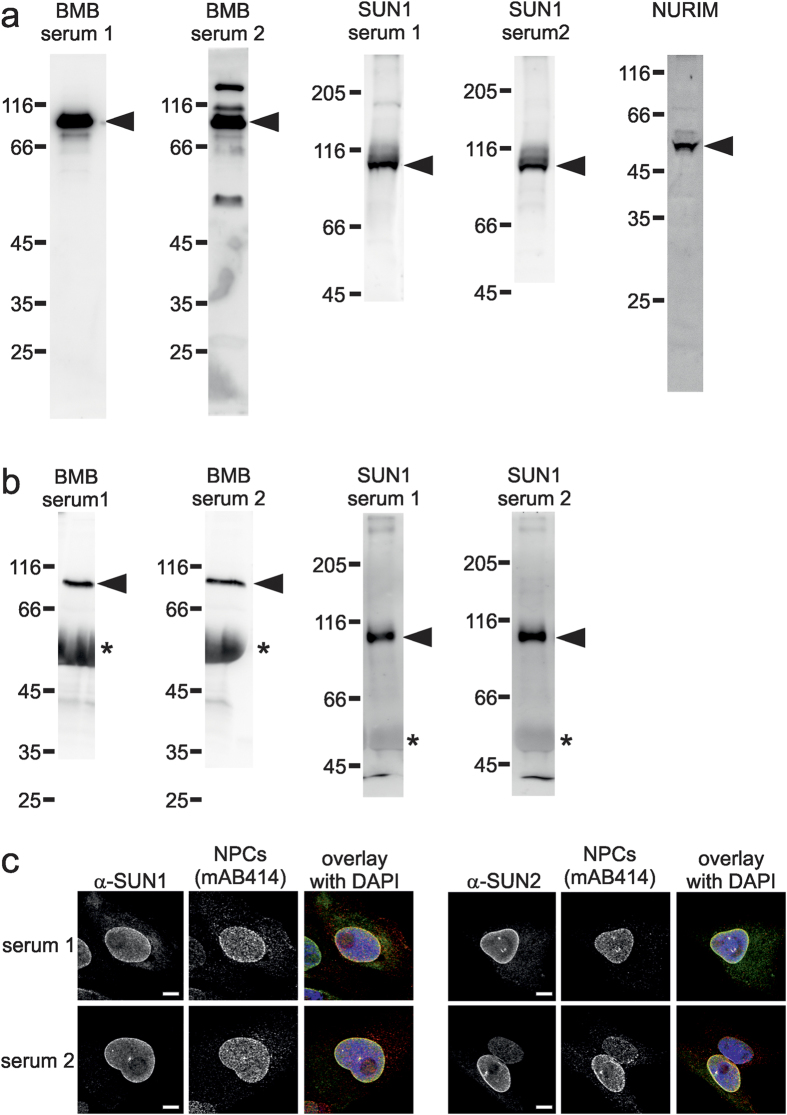
MISTIC-fusion proteins are efficient antigens for production of antibodies against membrane proteins. (**a**) Two antisera generated against *Xenopus* full-length brambleberry (BMB), two antisera against SUN1 and one antiserum against full-length Nurim were analyzed by western blotting in a 1:1000 dilution. 3 μg of a total membrane fraction from *Xenopus* egg extracts were separated on 12% (for BMB and Nurim) or 8% SDS-PAGEs (for SUN1). Molecular size markers as well as the position of respective proteins are indicated. Cropped images showing the whole respective lanes are shown. (**b**) BMB and SUN1 were immunprecipitated from a solubilized total membrane fraction from *Xenopus* egg extracts using antisera described in (**a**). Antibody bound proteins were eluated with SDS sample buffer, separated on a 12% (BMB) or 8% SDS-PAGE and analyzed with BMB serum 1 or SUN1 serum 1, respectively. Molecular size markers as well as the position of respective proteins (arrow head) and the IgG heavy chain (*) are indicated. Cropped images showing the whole respective lanes are shown. (**c**) Immunofluorescence detection of SUN1 and SUN2. *Xenopus* S3 cells were fixed with 2% PFA and stained with two antisera against SUN1 (as in a) and full-length *Xenopus* SUN2. Samples were co-stained with the nuclear pore complex (NPC) marker mAB414 and DAPI and analyzed by confocal microscopy. Bars: 5 μm.

To strengthen our hypothesis that MISTIC-fusion membrane proteins are efficient antigens for antibody production we raised antisera against a number of additional nuclear envelope proteins using MISTIC-fusion proteins. The target proteins were SUN1 and SUN2, inner nuclear membrane proteins and part of the LINC complex which connects inner and outer nuclear membrane (for review see ref. 26). Nurim is an ill-characterized inner nuclear membrane protein with six predicted transmembrane regions^27^. Brambleberry (BMB) is a membrane spanning protein of the nuclear envelope implicated in membrane fusion between nuclei^28^. We observed that all antisera obtained against BMB, SUN1 and NURIM were functional in western blotting (Fig. 5a). In addition, all antisera against BMB and SUN1 efficiently immunoprecipitated the respective target proteins from *Xenopus* egg extracts (Fig. 5b). SUN2 is not expressed in *Xenopus* eggs and is thus not included in panel (a) and (b). All antisera against SUN1 and SUN2 were functional in immunofluorescence labeling the nuclear envelope as expected (Fig. 5c). Thus, these data support the view that MISTIC-fusion proteins are excellent antigens for generation of antibodies against a wide range of membrane proteins.

**Figure 6:**
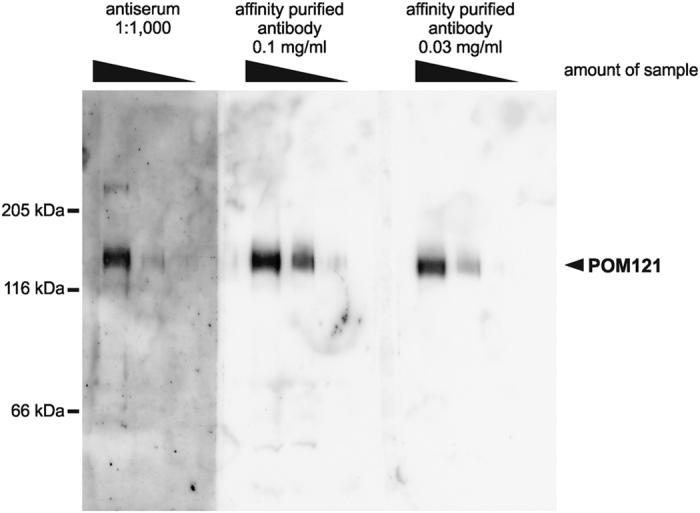
Antisera raised against MISTIC-fusion proteins can be efficiently affinity purified. An antiserum raised against a MISTIC-fusion with the full-length transmembrane nucleoporin POM121 was affinity purified using the MISTIC-POM121 protein coupled to Affigel 10 matrix as bait. The unpurified antiserum (at a 1:1,000 dilution) as well as the affinity purified antibodies (at a 0.1 mg/ml and 0.03 mg/ml dilution) were tested by western blotting after 6% SDS-PAGE of 10 μg, 3 μg and 1 μg of a total membrane fraction from *Xenopus* egg extracts. Molecular size markers as well as the position of POM121 are indicated.

Antiserum from an immunized rabbit can be directly used for a number of applications, but the antiserum contains in addition to the specific IgG recognizing the desired antigen a huge amount of other antibodies and other components of the serum. For some applications, purification of the antigen specific antibodies is desirable. For this, the respective antigen is often coupled to a gel material and used as an affinity matrix. All MISTIC-fusion proteins tested could be coupled to standard pre-activated affinity media such as CNBr-Sepharose or Affigel 10/15, which are often employed for such affinity purifications, and the respective affinity matrixes were used for purification of the specific antibodies. We show here as an example an affinity purification of an antiserum against the nuclear pore complex transmembrane protein POM121^29^, which has an essential function in nuclear pore complex assembly^30^,^31^. Affinity purified POM121 antibodies detect the target protein efficiently in western blotting even when employed in high dilution (Fig. 6). In addition, a crossreactivity detected with the original antiserum is now longer noticeable when using the affinity purified antiserum indicating an improved specificity.

**Figure 7:**
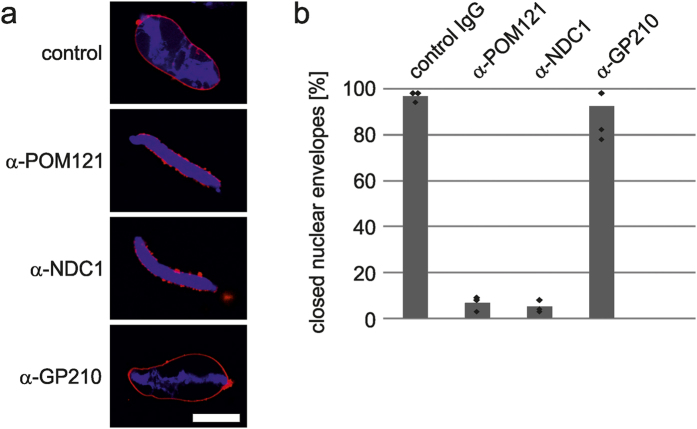
Antibodies raised against full-length POM121 and NDC1 inhibit nuclear reformation. (**a**) *Xenopus* egg extract membranes were preincubated with control or α-POM121, α-NDC1 or α-GP210 IgG, respectively, and used in nuclear assembly reactions. Membranes were stained with DiIC18 (red) and chromatin with DAPI (blue) and samples analyzed by confocal microscopy. As previously reported for antibodies against soluble domains of POM121 and NDC1^22^,^30^, also antibodies against full-length POM121 and NDC1 but not GP210 block formation of a closed nuclear envelope around sperm chromatin. (**b**) 100 randomly chosen chromatin substrates per reaction from samples from (a) were analyzed for closed nuclear envelope formation. The average of three independent experiments are shown, individual data points are indicated.

Next, we tested whether affinity purified antibodies generated against transmembrane proteins could be employed in a functional assay as an inhibitory reagent. We have previously shown that formation of a closed nuclear envelope at the end of mitosis requires two integral nuclear pore complex membrane proteins, POM121 and NDC1^18^,^25^. Nuclear envelope formation can be reconstituted in a cell free assay using *Xenopus* egg extracts^17^. Upon incubation of sperm DNA in cytosol and a purified membrane fraction from these egg extracts a closed nuclear envelope, detected by the smooth DiIC18 (1,1′-Dioctadecyl-3,3,3′,3′-Tetramethylindodicarbocyanine, 4-Chlorobenzenesulfonate) membrane staining, forms around the chromatin (Fig. 7a). When preincubating the membrane fraction with affinity purified antibodies against POM121 and NDC1 generated using the MISTIC-fused full-length proteins as antigens we observed an inhibitory effect (Fig. 7a and b). This is consistent with previous data, where we had observed the same inhibitory effect with antibodies generated against soluble fragments of POM121 and NDC1^18^,^25^. In contrast, antibodies against the third nuclear pore complex transmembrane protein GP210 did not show an inhibitory effect (Fig. 7a and b) as previously reported^30^. We conclude that as expected antibodies generated against MISTIC-fusion proteins can be used as inhibitory agents against transmembrane proteins in biochemical/cellular assays.

## Discussion

Antibodies are among the most frequently used and highly versatile tools in basic research and in clinical assays. These assays often combine an extraordinary sensitivity with outstanding selectivity when detecting the respective target molecules. Hence there is a constant demand for these reagents which is illustrated by the fact that the global market for research antibodies reached around $1.6 billion in 2011 and is still rapidly growing^32^. Antibodies against transmembrane proteins have traditionally been one of the most difficult research reagents to procure. The protocol presented here allows with only modest laboratory effort the efficient generation of high quality polyclonal rabbit antibodies. In functional assays the generated antibodies against the MISTIC-fusion membrane proteins were superior to antibodies generated against a soluble domain of the respective protein. The antigens used included single and multispanning membrane proteins. The method was successfully tested using different standard immunization additives as Freud complete/incomplete adjuvant or Titermax (see Materials and Methods). We observed a constant batch quality when bleeding an immunized rabbit for a period of up to 6 month (data not shown). Thus, the presented method expands the method repertoire for generating antibodies to the so far difficult targets of membrane proteins. Although all examples we present here are antibodies against *Xenopus* membrane proteins we also expect a significant increase in antibody quality for human or mouse membrane proteins using this strategy and will test this in future experiments.

MISTIC-fusion has been shown to enhance the expression levels of foreign integral membrane proteins in *E. coli.* Mostly, this has been compared to an only HIS6-tagged target protein^9^,^14^,^33^,^34^,^35^,^36^. Indeed, when testing this for one of our single membrane spanning test proteins, SCL1/BC08, we observe a similar major increase in expression of the MISTIC versus the only HIS6 tagged protein (see Supplementary Fig. S1). In addition, the MISTIC fusion shows also significantly increased expression yields as compared to other solubility tags as NusA or GST (see Supplementary Fig. S1). This is consistent with previous observations where the expression of the integral membrane metalloprotease HtpX shows among several solubility tags the best expression when fused to MISTIC^37^. The MISTIC-fusions are expected to be found in the pellet fraction after lysis^12^ and to be solubilized after detergent treatment. Indeed, using the detergent Cetyltrimethylammoniumbromid (CTAB) we observe good solubilization of the MISTIC-fusions from the pellet fraction (see Supplementary Fig. S2).

MISTIC-fusion membrane proteins might function as efficient antigens because within the transmembrane region additional epitopes can be presented to the immune system as compared to the antigens lacking transmembrane regions. Interestingly, the quality of the antisera significantly improved even when a single transmembrane region does not contribute a considerable fraction of the mass of the antigen. This is best illustrated for the antisera against SCL1/BC08 and Calnexin (Figs 3 and 4). We thus prefer the hypothesis that expression and purification of an intact membrane protein more likely preserves the native state of the protein in comparison to expressing only a soluble fragment and performs therefore a better antigen. Indeed, the N-terminal half of NDC1 comprising all six predicted transmembrane regions generates, in contrast to the full-length protein, not exceptional antisera (Fig. 2). Thus, it is unlikely that it covers a huge fraction of high affinity epitopes. Yet, antisera generated against the full-length protein are superior to ones against a soluble fragment of the C-terminal half of the protein.

Membrane proteins represent a large and growing class of drug targets^3^,^4^ as plasma membrane proteins are accessible to circulating drugs and membrane protein are often involved in regulating disease states. They are thus of primary interest in cell biological and biomedical research. Although tested here for the production of polyclonal rabbit antibodies, which is often the primary choice for antibody production in basic research, the method presented here to use MISTIC-fusion proteins as antigens is likely to be also applicable for the development of monoclonal antibodies and nanobody reagents and thus suitable in biomedical applications.

## Materials and Methods

### Expression and purification of MISTIC fusions

Transmembrane proteins were expressed from a modified pET28a vector (Novagen) containing a MISTIC sequence upstream and a HIS6 tag downstream of the expressed gene. The expressed proteins are full-length *Xenopus laevis* POM33 (aa 1–247, gene bank accession NP_001086534.1), full-length *X. laevis* NDC1 (aa 1–660, gene bank accession NP_001087436.1), full-length *X. laevis* POM121 (aa 1–1050, gene bank accession NP_001089069.1), full-length *X. laevis* NURIM (aa 1–285, gene bank accession NP_001085892.1), full-length *X. laevis* SCL1/BC08 (aa 1–97, gene bank accession KX588241), full-length *X. laevis* brambleberry (aa 1–644, gene bank accession KX588242) full-length *X. tropicalis* SUN1 (aa 1–873, gene bank accession XP_002941853.1) and *X. laevis* SUN2 (aa 1–709, gene bank accession BC086466.1). Similarly, a fragment of *X. laevis* calnexin (aa 485–611, gene bank accession NP_001080326.1) comprising the transmembrane region and the cytoplasmic domain and a fragment comprising the N-terminal part of *X. laevis* NDC1 (aa 1–301) which includes all six predicted transmembrane helixes was expressed.

Proteins were expressed in *E. coli* BL21de3 using an autoinduction protocol. *E. coli* precultures were grown over night in LB (lysogeny broth) medium containing 0.25 mg/l Kanamycin and diluted the next morning 1:100 in autoinduction medium. Autoinduction medium was prepared by supplementing LB medium with 0.25 mg/l Kanamycin, autoinduction solution (from 50X stock solution, final concentrations in medium 0.5% glycerol, 0.05% glucose, 0.2% alpha-lactose) and NPS buffer (from 20X stock solution, final concentrations in medium 50 mM KH2PO4, 50 mM Na2HPO4, 25 mM (NH4)2SO4). Cells were grown at 37 °C until an OD600 of approximately 1.5 was reached and cultures were subsequently shifted to the expression temperature of 25 °C. Protein expression occurred over night. The next morning bacteria were pelleted by centrifugation (3800 g, 15 min, 4 °C) and pellets resuspended in ice cold 200 ml per 1 l expression culture Ni wash buffer (20 mM Tris pH 7.5, 500 mM NaCl, 30 mM Imidazole pH 7.5). Bacteria were lysed by high pressure (900 bar) using an EmulsiFlex system (Avestin). Cell lysates were subsequently supplemented with 2 mM MgCl2, 2 mM PMSF and 20 mg/l DNase I (Calbiochem) and incubated for 10 min on ice. Soluble proteins were separated from membrane fractions by centrifugation at 30000 g for 20 min at 4 °C. All following steps were done at RT and buffer tempered at 20 °C. The membrane pellet obtained from 1 l expression culture was resuspended in 320 ml Ni wash buffer + 1% (w/v) CTAB (Calbiochem) and rotated for 1 h to allow membrane protein solubilization. Solubilized membrane proteins were separated from insoluble cell debris by centrifugation (15000 g and 20 °C for 15 min). The supernatant containing detergent solubilized membrane proteins was incubated with Ni-NTA-Agarose (Qiagen) and HIS6-tagged proteins were bound to the beads for 2 h. Ni-NTA-beads were collected in a chromatography column and washed with 50 bed volumes of Ni wash buffer + 0.1% CTAB. Proteins were eluted with Ni elution buffer I (20 mM Tris pH 7.5, 500 mM NaCl, 400 mM Imidazole pH 7.5, 1% CTAB) and dialyzed for 1 h and overnight in PBS containing 1 mM EDTA.

### Expression and purification of soluble domains

Protein fragments comprising soluble domains were expressed from a pET28a vector (Novagen) and include the C-terminal nucleoplasmic domain of *X. laevis* POM33 (aa 195–247), the C-terminal/cytoplasmic domain of *X. laevis* Calnexin (aa 516–611) and the nucleoplasmic domain of *X. laevis* SCL1/BC08 (aa 1–76).

Proteins were expressed in *E. coli* BL21de3 using the autoinduction method (see above). Bacteria were pelleted by centrifugation (3800 g, 15 min, 4 °C) and pellets resuspended in ice cold 200 ml per 1 l expression culture Ni wash buffer. Bacteria were lysed by high pressure (900 bar) using an EmulsiFlex system. Cell lysates were subsequently supplemented 2 mM MgCl2, 2 mM PMSF and 20 mg/l DNase I and incubated for 10 min on ice. Soluble proteins were separated from insoluble material by centrifugation at 30000 g for 20 min and the supernatant was incubated for 2 h at 4 °C with Ni-NTA-Agarose for binding of HIS6-tagged proteins. Ni-NTA-beads were collected in a chromatography column and washed with 50 bed volumes of Ni wash buffer. Proteins were eluted with Ni elution buffer II (20 mM Tris pH 7.5, 500 mM NaCl, 400 mM Imidazole pH 7.5) and dialyzed for 1 h and overnight in PBS containing 1 mM EDTA.

### Antisera production

Rabbit polyclonal antibodies were generated using standard immunization protocols. In short, for the primary injection 1 ml Freud complete adjuvans (Rockland) was mixed with 1 ml of antigen solution (1.0 mg/ml protein in PBS) and subcutaneously injected in four sites. 14, 28 and 56 days after the primary injection, rabbits were boosted by subcutaneous and intramuscular injection with a mixture of 1 ml Freud incomplete adjuvans (Rockland) and 1 ml antigen solution with a protein concentration of 0.5 mg/ml for the first and 0.2 mg/ml for the second and third boost. Blood samples were taken 10 days after the third boost and serum generated. When employing TiterMax^®^ Classic (Sigma) as an adjuvans, 2 ml of the protein solution (0.5 mg/ml, 0.25 mg/ml, 0.1 mg/ml, 0.1 mg/ml in PBS for the respective injections) were mixed with the adjuvans and injected at time points as above.

Antisera against a soluble fragment of *X. laevis* NDC1 (aa 361–521)^22^ and *X. laevis* GP210^30^ have been described before.

### Western blot analysis

Total *X. laevis* egg extract membrane fraction was heated for 5 min at 65 °C in SDS-sample buffer containing 20 mM DTT. Samples were separated using SDS-PAGE using the Mini-Protean system (Biorad) and blotted to a Protran 0.45 μm nitrocellulose membrane (Whatman). Proteins were transferred for 3 h at 4 °C using the wet transfer method in 2 mM TRIS, 15.4 mM glycine, 20% ethanol using Mini-Trans-Blot cells (Biorad). For the detection of SCL1/BC08 the semidry method was used with 48 mM TRIS pH 8.4, 39 mM glycine, 0,04% SDS and 20% methanol as transfer buffer using the Trans-Blot SD semi-Dry device (Biorad). After transfer the membrane was stained with a 0.1% (w/v) Ponceau S solution in 1% (v/v) acetic acid and the molecular size marker bands marked. Then, the membrane was blocked o/n at 4 °C with PBS-T buffer (PBS and 0.1% Tween 20) containing 5% (w/v) skim milk and rinsed with PBS-T buffer. The next day the membrane was probed for 2 h at RT with antisera if not otherwise indicated in a 1:1,000 dilution in PBS-T buffer with 5% (w/v) skim milk. After several washes with PBS-T buffer the membrane was incubated for 1 h at RT with goat anti-rabbit IgG-HRP (Calbiochem) in a 1:5,000 dilution. For western blot analysis of immunprecipitations Protein-A-HRP at a dilution of 1:2,000 was employed to minimize detection of the on the SDS-PAGE separated heavy chains of the IgG. After several washes with PBS-T buffer the membrane was quickly washed with bidestilled water and analyzed using Western BrightTM Quantum ECL (Biozym) on a Fusion Solo Vilber Lourmat (Peqlab). The FusionCapt Advance software was used for image analysis.

### Immunoprecipitations

Total *X. laevis* egg extract membrane fraction^17^ was solubilized in 1% Triton X-100 in PBS for 10 min at RT. Insoluble material was spun off by centrifugation at 500,000 g for 10 min in a TLA 100 rotor (Beckman). 800 μl of the supernatant was mixed with 100 μl of the respective antisera in a MoBiColumn “Classic” spin column (MobiTec) and rotated for 2 h at 4 °C. Then, 60 μl of the 50% Protein A-Sepharose (GE Healthcare) slurry was added and the sample rotated for another hour. Next, the column was spun for 30 s at 2,000 rpm in a cooled centrifuge and the flow through discarded. The remaining beads are washed five times with 70% PBS. The sample is eluted with 40 μl SDS-sample buffer and analyzed by SDS-PAGE and Western blotting.

### Immunofluorescence analysis

*Xenopus* S3 cells were grown in 70% Leibovitz L-15 media GlutaMAX Supplement, 10% FBS and 500 units/ml penicillin-streptomycin (all from Gibco). Cells quickly washed with 70% PBS supplemented with 0.1 (v/v)% Triton X-100 and fixed for 5 min in 2% paraformaldehyde supplemented with 0.5 (v/v)% Triton X-100. Immunofluorescence analysis was performed as described^17^. The rabbit polyclonal antibody was used in a 1:100 dilution and visualized by an Alexa 488-conjugated goat anti-rabbit antibody (Invitrogen) diluted 1:2,000. Mab414 ascites (Covance, MMS-120R), which marks nuclear pore complexes, was used in a 1:2,000 dilution and visualized by a Cy3-conjugated goat anti-mouse antibody (Invitrogen) diluted 1:2,000. Chromatin was stained with 10 μg/ml DAPI. Fluorescence images were acquired using a confocal microscope (LSM780, Zeiss) using 405-, 488-, and 561-nm laser lines and an apochromat 63 × NA 1.40 oil DIC M27 objective.

### Affinity purification

For coupling of antigens to Affigel 10 or 15 (Biorad) we followed the manufacturer’s instructions. In short, 1 ml Affigel 15 (for proteins with a pI < 6.0) or Affigel 10 (for all other proteins) matrix was washed with bidest water and incubated with 10 ml of the antigen (2 mg/ml protein concentration in 20 mM HEPES, 500 mM NaCl pH 7.4) for at least 4 h at 4 °C. After removal of unbound protein unreacted functional groups of the matrix were quenched with 3 ml 1 M ethanolamine pH 8.0 for 1 h at 4 °C. After two washes with 15 ml 100 mM NaHCO3, 500 mM NaCl pH 8.3, two washes with 15 ml 100 mM Na-acetate, 500 mM NaCl pH 4.2, one wash with 15 ml 200 mM glycine, 150 mM NaCl pH 2.3, and three times 15 ml PBS the resin is stored in PBS supplemented with 0.1% NaN3 and 0.1 mM PMSF till use.

For affinity purification 1 ml of the resin was incubated with 10 ml serum for at least 4 h at 4 °C. After three washes with 15 ml PBS supplemented with 0.1% Tween 20 and three washes with 15 ml PBS antigen bound antibodies are eluted with 3 ml 200 mM glycine, 150 mM NaCl pH 2.7 and 5 ml 200 mM glycine, 150 mM NaCl pH 2.3. The eluted antibodies were immediately neutralized with 1 M TRIS pH 8.8 and dialyzed to PBS. If necessary, antibodies were concentrated by precipitation with 6 M ammonium sulfate, resuspended in a small volume of PBS and dialyzed to PBS. Antibody concentrations of usually 1 mg/ml were obtained.

### Nuclear assembly reactions

Nuclear assembly reactions were performed as described in detail^17^. For inhibition experiments, 4 μl of floatation purified DiIC18 (1,1′-Dioctadecyl-3,3,3′,3′-Tetramethylindodicarbocyanine, 4-Chlorobenzenesulfonate) labeled membranes^17^ were preincubated with 4 μl of 1 mg/ml affinity purified α-NDC1, α-POM121, α-GP210 or unspecific rabbit IgG control antibodies dialyzed to sucrose buffer (10 mM HEPES, 50 mM KCl, 2.5 mM MgCl2, 250 mM sucrose). After 10 min incubation the membranes were used in a nuclear assembly reaction with 20 μl cytosol. After 120 min the samples were fixed, re-isolated (described in ref. 17) and analyzed by confocal microscope (FV1000; Olympus; equipped with a photomultiplier [model R7862; Hamamatsu]) using 405-, and 559-nm laser lines and a 60 × NA 1.35 oil immersion objective lens.

## Additional Information

**How to cite this article**: Alves, N. S. *et al*. MISTIC-fusion proteins as antigens for high quality membrane protein antibodies. *Sci. Rep.* **7**, 41519; doi: 10.1038/srep41519 (2017).

**Publisher's note:** Springer Nature remains neutral with regard to jurisdictional claims in published maps and institutional affiliations.

## Supplementary Material

Supplementary Material
