# Supplementary material for: MISTIC-fusion proteins as antigens for high quality membrane protein antibodies
Source: Sci Rep. 2017 Feb 2;7:41519. doi: 10.1038/srep41519 (PMC5288655; doi:10.1038/srep41519)
Supplement: Supplementary Material [file srep41519-s1.pdf]

Supplementary Information

MISTIC-fusion proteins as antigens for high quality membrane protein antibodies

Natalia Silva Alves (1), Susanne Adina Astrinidis (1), Nathalie Eisenhardt (2), Cornelia Sieverding, Josef Redolfi, Michael Lorenz, Marion Weberruss, Daniel Moreno-Andrés, Wolfram Antonin

(1) Equally contributed
(2) Current address: Max Planck Institute of Immunobiology and Epigenetics, Department of Epigenetics, Stuebeweg 51, 79108 Freiburg, Germany

Friedrich Miescher Laboratory of the Max Planck Society, Spemannstraße 39, 72076 Tübingen, Germany

wolfram.antonin@tuebingen.mpg.de

Supplementary Methods

For Supplementary Figure 1, full-length *Xenopus* SCL1/BC08 (aa 1-97, gene bank accession KX588241) was expressed from a modified pET28a vector (Novagen) containing no additional tag or a NusA or GST-tag sequence upstream and a HIS6 tag downstream of the SCL1/BC08 sequence in *E. coli* BL21de3 using an autoinduction protocol as described in the main text. In addition, the MISTIC-fusion described in the main text was used. Soluble proteins were separated from membrane fractions by centrifugation at 30000 g for 20 min at 4°C.

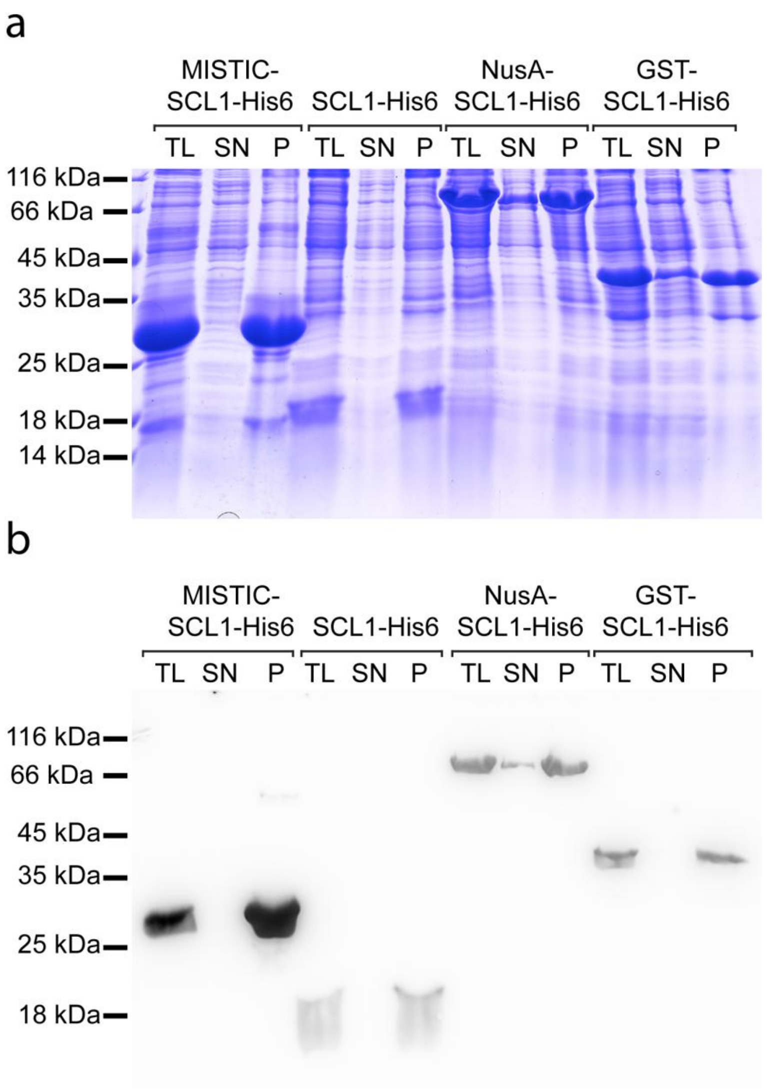

Supplementary Figure S1: expression of differently tagged SCL1/BC08 fusions

Full-length SCL1/BC08 was expressed as MISTIC, NusA and GST fusion, all with a C-terminal HIS6-tag or as only C-terminal HIS6-tagged protein in *E. coli*. After lysis of the cells, equal relative amounts of the total lysate (TL), the supernatant (SN) and the resuspended pellet (P) of a 30.000 g centrifugation of the lysate were analyzed by 15% SDS-PAGE followed by Coomassie Blue staining (a) or western blotting (b) using an α-HIS6 antibody (1:500 dilution, Roche, order number 4905318001). Please note that addition of the NusA or GST as solubility tags to this relative small single membrane region containing protein increases expression yields. However, addition of the MISTIC tag outperforms even the large NusA tag.

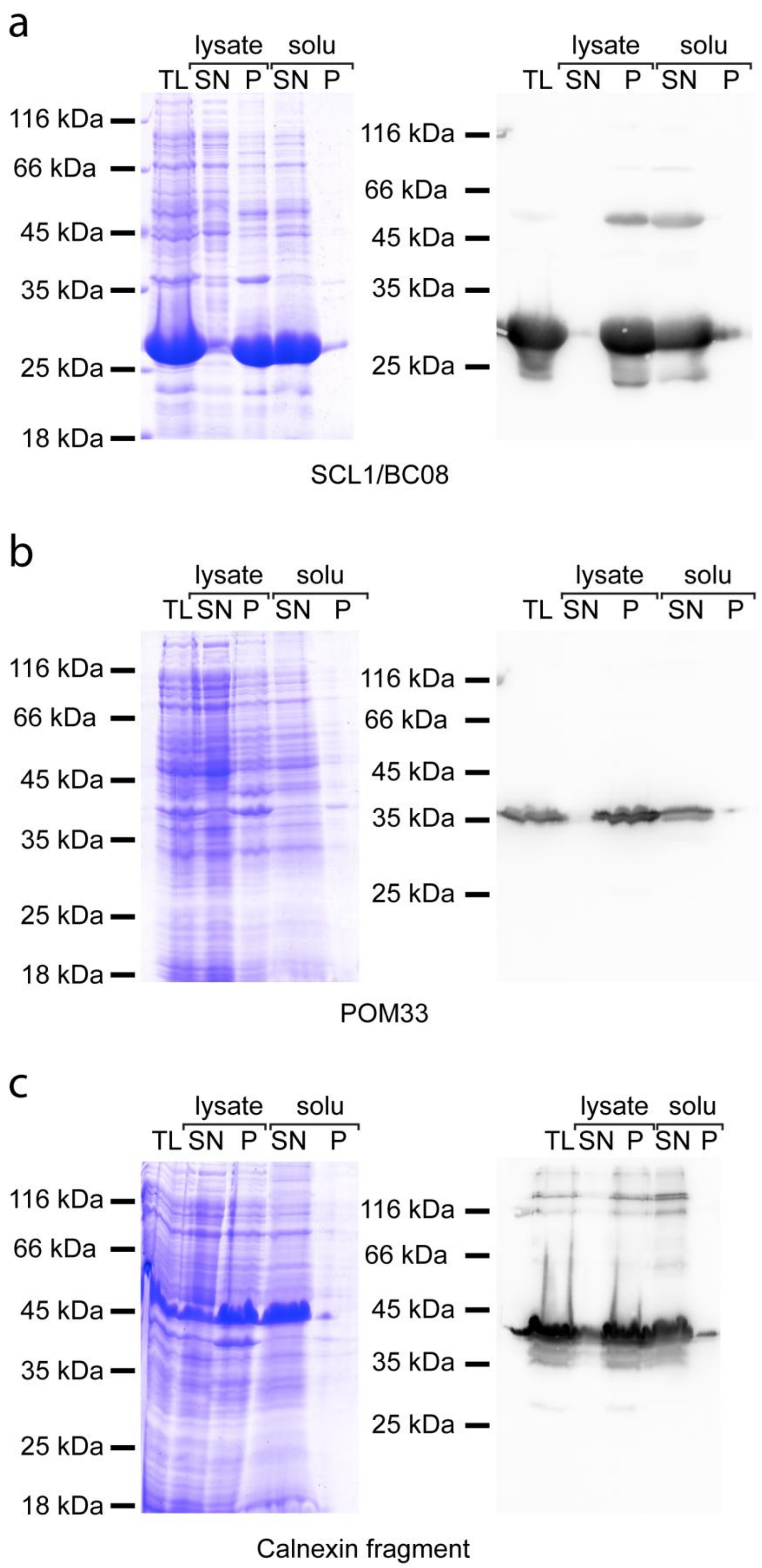

Supplementary Figure S2: expression and solubilization test for different MISTIC fusions

Full-length SCL1/BC08 (a), POM33 (b) and a Calnexin (c) fragment (aa 485-611) were expressed as MISTIC-fusion in *E. coli*. After lysis of the cells, equal relative amounts of the total lysate (TL), the

supernatant (SN) and the resuspended pellet (P) of the 30.000 g centrifugation of the lysate as well as the supernatant and resuspended pellet of the 15.000 g centrifugation after solubilization (solu) were analyzed by 12% SDS-PAGE and Coomassie Blue staining (left) and western blotting using an α-HIS6 antibody (1:500 dilution, Roche, order number 4905318001, right panel). Please note efficient solubilization from the lysate pellet fraction for all three constructs.
